# Entomophagy: A Narrative Review on Nutritional Value, Safety, Cultural Acceptance and A Focus on the Role of Food Neophobia in Italy

**DOI:** 10.3390/ejihpe10020046

**Published:** 2020-06-03

**Authors:** Elisabetta Toti, Luca Massaro, Aisha Kais, Paola Aiello, Maura Palmery, Ilaria Peluso

**Affiliations:** 1 Research Centre for Food and Nutrition, Council for Agricultural Research and Economics (CREA-AN), 00142 Rome, Italy; msluca96@gmail.com (L.M.); aishakais@libero.it (A.K.); ilaria.peluso@crea.gov.it (I.P.); 2 Department of Physiology and Pharmacology “V. Erspamer”, Sapienza University of Rome, 00185 Rome, Italy; paola.aiello@uniroma1.it (P.A.); maura.palmery@uniroma1.it (M.P.); 3 Faculty of Health Sciences, Universidad Católica San Antonio de Murcia, Murcia (UCAM), 30107 Murcia, Spain

**Keywords:** consumers’ acceptance, food safety, food attitude, edible insects, Italy

## Abstract

In recent years, the consumption of insects, or entomophagy, has produced an increasing interest amongst scientists and ecologists as a potential source of animal protein. Eating insects is also interesting in terms of low greenhouse gas emissions and low land use. In contrast to tropical countries, where most of the 2000 edible insect species are traditionally consumed, the concept of eating insects is still new to Western culture and diet. Culture and eating habits exert a great influence on what is considered edible in the Mediterranean area, especially in Italy, where the preservation of culinary traditions is a predominant factor affecting dietary behaviour. The purpose of this narrative paper is to provide an overview of the main topics related to entomophagy. The introduction presents some information about the nutrient content and safety aspects, the second part summarises the cultural acceptance of insect in the world, while the role of food neophobia on the intention to consume insects in Italy is focused on in part three. The discussion displays important viewpoints of previously published studies and based on these perspectives it can be concluded that the Italian diet is still clearly influenced by local tradition. In conclusion, in order to introduce insects into the Italian diet, psychological motivation has to be enhanced.

## 1. Introduction

Edible insects have been suggested as a source of proteins, amino acids, essential fatty acids, fibre and micronutrients [1,2]. Insects are environmentally friendly, as they can recycle waste, require little food and water for their growth, and have a rapid growth rate [3,4]. For all these reasons, in recent years, entomophagy has reached global attention and currently the potential use of insects as a new source of food for humans appears extremely interesting [5]. It has been suggested that insects could be a promising alternative source of animal protein with a reduced environmental impact [6,7], however, further research is needed to verify any possible risk to human health. Legislation on production, transformation and commercialization aspects have been previously reported by other authors in some review articles [5,8,9]. In Italy, an informative note, published on 8 January 2018 by the Italian Health Ministry, clarified that insects belong to a group of novel foods, which have not been authorized yet [10]. The absence of Italian legislative authorization and the precautionary principle asserted in the Official Journal of the European Union (EU Regulation 2015/2283) are not the only factors that explain the attitude of Italian consumers towards entomophagous practices [11]. In fact, psychological, sociological and anthropological factors play an important role in the choice of insect products by Italian consumers [12].We aimed to make an overview on some aspects of entomophagy and in particular, discuss the existing aversion amongst Mediterranean consumers, with particular reference to the Italian population, in which complex food decision-making, due to attachment to tradition, has been identified [2].

### 1.1. Nutritional Value

Providing a complete worldwide picture on the edible insect species is very complex because there is limited information on species eaten as part of traditional diets in different geographical areas. Out of the millions of insect species living on Earth, about 2000 are considered edible and their use varies between regions and cultures [13,14]. According to Jongema (2017) [14], most of the insects consumed belong to the order of Coleoptera (e.g., beetles), followed by Lepidoptera (e.g., caterpillars), Hymenopterans (e.g., bees, wasps and ants), Orthoptera (e.g., grasshoppers, locusts and crickets), and lastly, Hemiptera (e.g., cicadas, leafhoppers, cochineals and bugs). Studies on the nutritional composition of edible insects should meet internationally recognized standards. The variation in nutrient composition is due to different analytical methodologies, the heterogeneity existing between insects from different environments and different rearing [15,16] and storage conditions [17]. The international network of food data systems (FAO INFOODS) has recently published a database on food composition [18] and in version 4.0 a total of 471 edible insects, with different methods of preparation, have been included [19]. Energy, macronutrient, micronutrient and fibre content, from the FAO INFOODS database, are summarized in Table 1. 

The protein content of insects ranges from 7% to 68% (Table 1) and in current literature, huge declines in the edible insects’ protein content during processing have been identified [20,21]. Non-protein nitrogen (NPN) in insects (chitin, nucleic acids, phospholipids, as well as ammonia in the intestinal tract) could lead to an overestimation of the protein content [22]. For this reason, instead of the conversion factor (Kp) of 6.25 generally used for proteins, a Kp of 4.76 has been suggested for whole larvae (from *Tenebrio molitor*, *Alphitobius diaperinus* and *Hermetia illucens*) and 5.60 for protein extracts derived from the larvae of the three insects studied [22,23]. 

Moreover, although some studies on rats have shown that *Acheta domesticus* cricket could be a good source of protein compared to vegetal sources (e.g., soy) [9,14], proteins from most insect species have limited amounts of tryptophan and lysine and their digestibility has been estimated between 77% and 98% [24,25]. 

Table 1 also reports the less saturated fatty acids, more monounsaturated and more polyunsaturated fats compared to meat total fat content present in edible insects, ranging from 1 to 57 g/100 g. Ramos-Elorduy et al. (1997) [25] reported that larvae and some adult insects with a soft body, such as termites, have the highest levels of fat, whilst insects with a hard exoskeleton, such as crickets and grasshoppers, contain smaller amounts. In terms of nutritional quality, the fatty acid composition is generally comparable to that found in poultry and fish, but insects probably contain less saturated fatty acids, more monounsaturated and more polyunsaturated fats compared to meat [1,2].

The fibre content of insects ranges from 1 to 15 g/100 g (Table 1) and is mainly sourced from the exoskeleton of chitin [26]. Although chitin could have positive effects on the immune system [5] and cholesterol levels [27], it has been suggested that chitin removal could improve the digestibility of insect proteins [22].

Micronutrient composition is highly variable and depends on the species of insect. A good source of iron is *Mopane* worm, with a content that reaches 508 mg/100 g [18]. In many species, the iron content is equal or greater than the iron content in beef [28]. For example, grasshoppers contain 8–20 mg/100 g of iron, whilst the iron content of beef is only 6 mg/100 g [13]. However, since iron absorption in humans is particularly complex and the iron compounds of insects are very different from those found in vertebrates [29], an assessment of the bioavailability, by means of human studies, is needed. 

Insect *larvae* also contain a great amount of zinc (98 mg/100 g) [18]. Recent literature reports that the domestic cricket (*Acheta domesticus*) contains 29.7 mg/100 g of zinc, whilst the domestic fly (*Musca domestica*) contains 85.8 mg/100 g [21]; values much higher than zinc levels in meat (an average of 12.5 mg/100 g in beef). 

Edible insects are also a significant source of vitamins, especially water-soluble vitamins such as B-vitamins. The Yellow Mealworm (or Flour Mealworm, *Tenebrio molitor*) is rich in vitamin B2, B6, B9 and B12, although the latter seems to be present only in *Tenebrio molitor* and *Acheta domesticus* [1]. Insects are not good sources of vitamin A, whereas commercially reared insects contain high levels of carotenoids [13].

### 1.2. Safety

The use of insects as food has many potential risks [30]. Although several studies have confirmed that levels of oxalate, phytic acid, phenol and tannins in edible insect species were below the toxicity levels for human consumption [31,32], a recent study suggested the possible bioaccumulation of methylmercury (MeHg) in dragonflies [33]. Furthermore, the concentration of exogenous substances (pesticides, lipophilic pollutants, drug residues and other bio-accumulative substances) depends on the metabolic characteristics of the insect and on the rearing methods [9]. Insect production may require pharmacological treatments to counteract possible infections and antibiotics, fungicides and anti-protozoan drugs can be used as possible treatments. The Hazard Analysis Critical Control Points (HACCP) and pre-requisites program (PRP) for insect production, storage, transport and labelling have been recently suggested [34].

The potential of insects to be carriers of pathogens such as protozoans (*Entamoeba histolytica*, *Giardia lamblia*, *Toxoplasma spp*. and *Sarcocystis spp*.) [35] and trematoda (*Dicrocoelium dendriticum)* is also a potential risk [36]. Viruses, such as *Arboviruses* can replicate in insects, in particular in flies and ticks, and can also infect humans [37]. *Arboviruses* cause diseases such as Dengue, Chikungunya, West Nile Disease, haemorrhagic fever, and Rift Valley fever [37]. However, there is no evidence that these viruses are present in edible species [38]. Moreover, viruses are vulnerable to food processing [38].

Arthropods can induce allergic reactions in humans, due to the presence of tropomyosin (contained also in shellfish and house dust mites), arginine kinase, glyceraldehyde 3-phosphate dehydrogenase, hemocyanine and hexamerin 1B [9,39,40]. Possible cross-reactions to crickets in individuals with a known allergy to crustaceans have been reported [39]. 

The role of arthropods (such as the *Musca domestica* and *Alphitobius diaperinus*) as vectors of *Salmonella* and *Campylobacter* is widely demonstrated [22]. *Casu Marzu*, a Sardinian cheese containing living larvae of the fly *Piophila casei*, is one of the most disputable cheeses in regards to safety issues in Europe, and Article 14 of Regulation (EC) No 178/2002 states that food shall not be placed on the market if deemed unsafe.

The results of national assessments conducted by authorities in Belgium, the Netherlands and France [41,42,43], have shown a high presence of aerobic and anaerobic bacteria in the Yellow Mealworm (*Tenebrio molitor*), locusts (*Locusta migratoria*) and in the larvae of the Giant Mealworm Beetle (*Zophobas atratus*). The process of roasting did not result in the total elimination of *Enterobacteriaceae* [44], whereas boiling at 100 °C for 8 minutes reduced the total aerobic bacterial count and the amount of *Enterobacteriaceae* to < 10 cfu/g [42]. It is also possible to reduce the total aerobic bacterial load and the amount of *Enterobacteriaceae* present in Mealworm larvae (*Tenebrio molitor*) and house crickets (*Acheta domesticus*) by drying the insects in an oven for 11 minutes at 90 °C [45]. The combination of high hydrostatic pressures (600 MPa) and high temperatures (90 °C) also reduced the bacterial count [1,46]. 

EFSA (2015) examined the potential risk related to the production and consumption of insects as food [38]. The microbial risk of edible species was found to be comparable to that of other animal protein sources [38], however, insects are commonly considered harmful by many consumers and the pursuit of pleasure, the preservation of traditions and the use of local and regional food are all known to affect food choices [47]. 

## 2. Entomophagy Versus Disgust in the World

**Figure 1:**
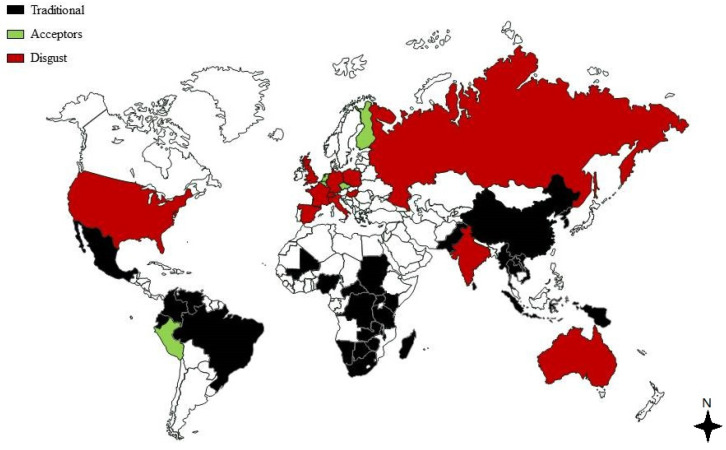
Map of disgust, acceptor and traditional countries [13,48,49,50].

In many countries the consumption of insects is part of the culture and tradition and, according to the FAO, insects are a common food source [13]. Insects are currently consumed as part of the daily diet in many developing and non-developing countries, including Africa, Asia, Latin America and Oceania (Figure 1).

Insects consumed in developing countries are currently collected in the wild, so their stage of development (larval or adult) and their availability are strictly dependent on seasonality [51]. In Africa, insects can be found throughout the continent and, particularly during the rainy season, the availability of caterpillars may vary even within the same country according to climatic conditions [51]. In Asia, the palm weevil (*Rhynchophorus ferrugineus*) of the sago palm (*Metroxylon sagu*) is popular throughout the continent and considered a delicacy in many regions [51]. Edible insects are used also in the Lao People’s Democratic Republic, Myanmar, Thailand and Vietnam [22,52]. Furthermore, over 50 species of insects are consumed in South Asia, 39 species in Papua New Guinea and the Pacific Islands, and between 150-200 species in Southeast Asia [51]. 

In the last fifteen years, Thailand has produced an average of 7500 tons of edible insects each year, including crickets, red palm weevils and bamboo caterpillars [53]. In Latin America, amongst the indigenous populations of Mexico and Brazil, there is a deep knowledge of the different species of insects that are traditionally part of their diet (Figure 1) [22]. Examples of traditional Mexican dishes are escamoles (the eggs of *Liometopum apiculatum Mayr*)*,* larvae of *Lepidoptera* and *Hemiptera* [22,54], chapulines (*Sphenarium purpurascens*) and chicatanas (*Atta mexicana*) [55]. Furthermore, entomophagy was extremely common amongst Australian Aborigines in the last 200 years, but the consumption of insects has decreased significantly due to the increasing adoption of European diets [22]. In fact, associating insects and diets is often stereotyped as a hallmark of an underdeveloped country or society, however, even in Europe, some types of insects are consumed. Parts or products of insects are eaten as raw snack food in some areas of the Friuli region (North Eastern Italy) such as the ingluvies of adult Lepidoptera (*Zygaenidae Zygaena spp*. and *Ctenuchidae Syntomis spp*.) [56]. *Casu Marzu* a cheese that contains live insect larvae, has a regional identity and is obtained using the milk of goat or sheep in Sardinia. Other examples of these cheeses can be found in other Italian regions including Friuli (*Saltarello* cheese) and Abruzzo (*Marcetto* cheese), and many others, such as *Gorgonzola delle Grotte* and *Formaggio di Fossa*, also exist. Similar cheeses are found in Corsica (France) and Croatia, as well as the German *Milbenkäse* or the French *Mimolette* [56].

A large number of surveys, focusing on European consumers, have shown that the propensity to consume insects as a meat substitute is generally low [50,57,58]. Moreover, participants who have previously eaten insects are more likely to eat them again [59,60]. In Holland, typical Dutch dishes such as burgers, nuggets and pittige punten (a spicy triangular product, similar in appearance to a hash brown or potato croquette), which are usually made from meat, were produced using vegetables and the ground larvae of the beetle *Alphitobius diaperinus* [47]. Consumers stated that the larvae were not visible, the taste of insects was not particularly identifiable and that the products were cooked similarly to conventional vegetarian foods. In this study, repeated consumption of insect-based products was relatively low (58% testing them only once; 18% more than once but not regularly; 24% semi-regular consumption; around 3% once every two weeks, weekly or twice a week) [47]. Initial reasons of consumption were dictated by a general interest or curiosity, by the feeling that insects are more environmentally friendly or sustainable than conventional meat-based products and by the belief that they are an alternative source of protein for a healthy diet. The participants’ general dietary guidelines had some influence with the preference for organic food being commonly reported amongst participants (mentioned by 42% of the group) [47]. 

Pambo et al. (2018) examined how consumers assess the appropriateness of sensory attributes of edible insects. The type of information that consumers received about the production process influenced the sensory assessment after tasting [61]. Another factor that could improve the acceptance of insects is the use of evocative names for insect recipes, which would attract attention and generate a good consumer expectation [62]. In addition, Castro and Chambers [63] suggested that insect-based product should not contain visible insect pieces, which trigger negative associations. In particular, the addition of cricket flour to traditionally consumed foods, could be an attractive option to introduce insects into diets without altering eating habits [22]. On the other hand, informing consumers about the reduced environmental impact deriving from the consumption of insects, can represent another strategy to increase insect consumption [20,60,64].

Biological, psychological and socio-cultural factors are known to influence food choices. Culture, in particular, influences what is considered edible, with many people in Western countries rejecting the idea of entomophagy mainly for cultural reasons. In a multi cross-cultural international survey including 630 individuals per country and representing 13 different countries (USA, Mexico, Peru, Brazil, UK, Spain, Russia, India, China, Thailand, Japan, South Africa, and Australia), authors identified “disgust” and “acceptor” countries (Figure 1) [48,63]. Compared to the UK [49], the likelihood of eating insect-based protein sources was more than twice in the Netherlands and Finland and 1.5 times in Spain. Willingness to try insect-products was higher in Mexico (71%), Peru (58%), Thailand (56%), Brazil (45%) and China (44%), but surprisingly lower in Japan (21%), where insects are part of the traditional diet and wasps are considered a highly sought-after food. Other countries had intermediate values (32%–36%) [48]. Although there are still several species that are eaten and considered a delicacy in Japan [65], a low willingness to try new-foods was found, probably due to the Japanese traditional diet [48]. The Japanese and the Mediterranean diet are considered healthy eating models [66,67,68], and this probably increases the attachment to eating traditional. The Mediterranean diet is included in the UNESCO Representative List of the Intangible Culture Heritage of Humanity [2] as it is a cultural product in an anthropological sense, and a lifestyle based on the conviviality of meals. For instance, food may enhance family unity when members consume it together [69]. Whilst the traditional food “Italian zampone” has been avoided in the Food Disgust Picture Scale (FDPS) [70], food “neophobia” (the fear of new or unfamiliar foods) [71] is associated with distaste [72] and could be higher in populations with strong traditional eating habits.

## 3. The Role of Food Neophobia on the Intention to Consume Insects in Italy 

Sixteen reviewed studies (from 19 publications), involving Italian individuals (Table 2), identified: food neophobia scale (FNS), willingness to try (WTT) or intention (ITE) or willingness to eat (WTE) or willingness to consume (WTC), willingness to pay (WTP), willingness to buy (WTB) and entomophagy attitude questionnaire (EAQ). Several definitions have been proposed to describe the intention to consume insect as food and in the light of these different definitions, it should not surprising that consumer intention for insects as food has been described in diverse way (WTT, WTE, WTC and ITE). 

FNS was described by one third of the studies (Table 2). One study involved 88 subjects aged between 18 and 40 years, and included students and staff (43 males and 45 females) [11,80,81]. The participants came from different geographical areas of Italy (20% North East, 36% North West, 14% Central Italy and 30% Southern Italy) and the questionnaire included the FNS and WTT. At the end of the questionnaire, two insect-based products (two sweet jellies with visible or processed cricket) were tasted. The results confirmed that the intention to taste is the most decisive factor for predicting the behaviour of consuming a new insect-based product. This intention is significantly determined by food neophobia and males were more WTT new foods [11,80,81]. In this study 75% of participants tasted the products and, concerning the tasting session of the reviewed studies (Table 2), percentages between 23% and 94% of the individuals (in the majority of the studies University students) were reported.

An online survey aimed at 3556 Italian university students aged 18–29 years old, found that 38% of the respondents were prone to consider that this food could be part of Italian diet. Reasonably, insect-food could be offered in a snack (where they would not be immediately recognizable) as a complementary source of proteins, considering that demand for proteins cannot be totally satisfied by the traditional livestock industry [74].

A study that evaluated WTE different insect-based food (cheddar cheese larvets, lollipops, chocolate covered scorpions, salt infused with chili and agave worm, dried crickets, baked grasshoppers and toasted scorpions) found that WTE was dependent on the form in which the products were presented [75]. 

Another analysis regarding Italy [76] showed that not only curiosity but also a focus on environmental benefits might be motivating factors to promote entomophagy amongst Italian consumers. The belief that insects have positive effects on the environment and relatively healthy and nutritious, increases the level of acceptance. Curiosity is also reported as a strong motivating factor [76] (Table 2) in fact, it was found to be one of the most significant drivers for acceptance in Southern Italy, whereas disgust and food neophobia were related to low acceptance [86]. One study, conducted in 2015 on a sample of 45 consumers (24 females and 21 males, aged between 24 and 39 years, students or just graduates), revealed the major barriers to the acceptance of insects as food are low familiarity with insect-based ingredients, neophobia, and/or visibility of insects in the product [86]. A soft laddering interview (free conversation) was performed to discover the personal values linked to attributes of the product which were perceived as benefits by the consumer. Interviews were conducted in the area of Naples. Kelly’s repertory grids (1955) were used and each consumer was shown three different imaginary products, similar to those already available in international online stores. From this study, it seems that curiosity is one of the most significant drivers for acceptance [86]. In another study conducted in South Italy, FNS significantly correlated with intention but not with disgust and the latter correlated significantly with intention [84]. 

The fear of insects and the idea that the taste might be disgusting, were the main barriers to the WTT entomophagy in Central Italy (Viterbo) [83] and gender also influenced consumer attitude (Table 2). On the other hand, no gender impact was observed in a recent study conducted at the University of Pisa (Italy) amongst students attending a seminar titled “Insects as Food and Feed: Future Prospects” [82]. Disgust factor decreased after the seminar, but volunteers indicated that they were less likely to use the "insect-labelled" bread, which was claimed to be supplemented with insect powder, in the future, despite the higher overall liking. The perception of positive effects on health had a stronger influence on behavioural intentions when compared to beliefs about environmental protection and familiar taste.

Despite bad taste being an important barrier to acceptance, disgust factor and the fear of negative texture properties were strongly reduced after the seminar generating a lower rejection. 

WTT was positively affected by behavioural intention. Students from South Italy [85] considered insect-based products either equivalent (the same WTP for the two versions of pasta) or slightly inferior (lower WTP in the case of cookies and chocolate) without information, whilst with information on benefits, consumers’ WTP increased for all the products. 

Another study based in Italy, investigated the role that product attributes can have in driving the perceptions of consumers of insect-based products. To a sample of 135 individuals were shown a series of products cards describing the products and they were asked to express their opinion. All the respondents reacted more positively to products made out of insect flour compared to the ones made with whole insects. Moreover, there were no incisive differences between opaque and transparent packaging. Neither the effect of cacao flavor nor the high-protein claim were statistically significant. Finally, the effect of the environmental certification appears to be not important in the food decision [87]. In a study conducted in an international university (trilingual: English, German and Italian), despite using persuasion strategies (including grounding insects into flour, disguising insects with cocoa, other peers’ reassuring statements on food safety, pleasant taste and availability) which positively influenced WTC [12], FNS negatively influenced both WTC and persuasion strategies [12].

In another study conducted in North Italy on 109 university students [77], more than two thirds of the subjects indicated they would taste edible insects if they had the opportunity and about half of the sample believed that entomophagy might become a culinary trend in Italy. However, high levels of education were shown to positively influence consumer attitude towards entomophagy [73,83]; therefore, opinions of university students could not be taken as being representative of the general population. 

Additionally, from a study conducted in North Italy and involving university students, employees and consumers from outside the university (223 females and 118 males—aged between 18–80 years), it was observed that younger people more readily accepted insects [73]. The authors, by grouping individuals according to FNS “low neophobia” (FNS scores ≤ 23), “medium neophobia” (FNS scores ≥ 24 and ≤ 41) and “high neophobia” (FNS scores ≥ 42), found a relationship between FNS and willingness to incorporate insects into diets and that environmental and nutritional benefits marginally affected the visual acceptability of insect-based foods. [73]. Although communication of both societal and individual benefits increased ITE, it has been reported that Danish individuals had higher ITE than Italians [60]. By using the EAQ (EAQ-I; EAQ-D; EAQ-F) differences were found between the Danes and Italians [88]. The EAQ includes three conceptual scores: disgust (EAQ-D), interest (EAQ-I) (including curiosity) and feeding animals (EAQ-F). The latter comprises the following sentence: “Using insects as feed is a good way of producing meat and I think it is fine to give insect-based feed to fish that are farmed for human consumption”. In the Danish volunteers, EAQ-I was the main predictor and regression coefficient EAQ-D vs. WTE insects, was much smaller than that of EAQ-I. A negative relationship of EAQ-F vs. intention toward direct entomophagy was found only in the Danish population. Amongst Italians no such difference between predictive power of EAQ-D and EAQ-I on WTE was found [88].

## 4. Discussion and Conclusions

Entomophagy is common in some Asian, American and African countries, whilst it is generally rejected by Western populations [12] and is often considered a “barbarian” tradition by Western culture [11]. Western food taboos, such as entomophagy, could be encouraged by cultural aspects in which insects are considered noxious products [89]. Western society generally regards insects as a food of emergency, not only associated with low prestige and indigent countries [76], but also with filth, danger or the psychological idea of possible contamination [72]. The Western reluctance towards entomophagy should not be classified as a mere form of disgust but as a form of acquired distaste derived from a lack of habits, and exposure not only to the flavour of insects but also their visual, tactile, olfactory and auditory properties and their sensory representation on dishes. Distaste has been defined as ’a form of motivated food rejection triggered by the ingestion of unpleasant tasting substances, prototypically those that are bitter’ [72], whose function is to avoid the ingestion of toxic compounds. Although it has been reported that curiosity is a strong motivational factor for trying insects [72], Italians tend to follow a diet based on the protective Mediterranean model [2].

Leon Rappoport analysing the social and psychological components of food claims: ’Consciously or not, when we eat we swallow not only a certain alimentary product, but also the concept, the culture, and the land to which it is associated with’ [90]. It has been suggested that rational theoretical assumptions encouraging "healthier" alimentary choices via prescriptive and legislative measures (e.g., sugar tax) are not the optimal strategies in the Italian context [2]. In Italy it has been reported that the belief of positive effects on health has a stronger influence on behavioural intentions than beliefs about environmental protection [82]. The potential success of a strategy in a country might be not suitable for another one. For example, the UK sugar tax may improve public health [91] but it seems not to be a useful strategy in Italy [2]. Briggs (2016) stated: ’Agriculture is responsible for up to 30% of the world’s greenhouse-gas emissions, yet it is often overlooked in climate discussions and was barely mentioned at December’s United Nations climate talks in Paris. Taxing food that is responsible for high greenhouse-gas emissions when it is produced and transported could benefit the health of both people and the planet. Sugar is a good start, but we can aim higher’ [91]. These sentences should make people engaged in health promotion campaigns of agri-food products and could alarm Italians, particularly tied to Mediterranean culinary traditions. It has been suggested that in Italy traditional values, such as the Mediterranean diet, might reduce the diffusion of genetically modified organisms-based foods [92].

Despite organic aquaculture might be a new and important strategy for diversification and labelling/certification are not taken into consideration, the added value of the production method might not be perceived by the final consumers that show a higher WTP for the sea bass country of origin than for the breeding method used [93]. Moreover, local origin geographic origin of honey accounted for 72.9% of the log-likelihood, followed by price and organic production [94]. 

Geographic origin is important also when choosing bovine meat and celebrating “Protected Geographical Indication” (PGI) Italy ranked first for an anniversary and a meal with friends [95].

In a study that explored consumers’ attitude towards cultured meat in Italy, people from northern Italy showed a significantly more positive perception towards attributes such as safety and sustainability than respondents from central and southern Italy [96]. In Italy the Sardinian sheep milk cheese “*Casu Marzu*” is well known and eaten, as well as other regional cheeses and some parts of *Lepidoptera* in Northern Italy are eaten [56]. In the present review, studies have been conducted in North [12,73,75,76,77,78,79], Central [82,83] and South Italy [84,85,86] and two studies involved volunteers from different Italian regions [11,74,80,81]. However, more research needs to be carried out to evaluate the effect of cultural variations exist among Italians of North, Centre and South on food neophobia and insects’ acceptance.

In conclusion, in order to introduce insects into the Italian diet, psychological motivation has to be enhanced. 

## Figures and Tables

**Table 1:** Nutrient composition of some edible insects (per 100 g edible portion on fresh weight).

	Coleoptera	Lepidoptera	Hymenoptera	Orthoptera	Hemiptera	Isoptera
Energy (kcal)	78–155	358–361	79–184	89–227	63–165	93–535
Protein (g)	13–21	49–55	7–14	13–68	19–20	21–21
Fat (g)	1–19	4–22	3–13	1–43	2–57	2–42
CHO (g)	1–3	12–18	5–6	1–5	3–8	20–21
Fiber (g)	5–7	4–15	1–3	2–10	4–5	5–6
Fe(mg)	0.3–24	0.03–109	3–103	0.1–42	0.4–29	0.1–31
Zn (mg)	5–6	2–11	4–15	4–13	4–46	3–8
Vit A (IU)	8–27	4.3–4.4	-	21–25	21–150	03–0.7
Vit E (IU)	0.7–1.2	8.3–8.6	-	1.0–2.3	1.4–13	0.8–1.0
Thiamine (mg)	0.2–0.3	-	0.2–0.3	0–0.4	0–0.6	-
Riboflavin (mg)	1.1–3.5	-	0.2–0.9	0.4–3.4	0.9–1.5	1.5–4.2
Vit.B12 (mcg)	-	-	-	5–9	-	-

From: FAO/INFOODS [18].

**Table 2:** Studies involving Italian individuals.

Sample (n)	Design	Major Results	Ref.
North Italy (Bolzano*): International university students (n.125), (trilingual: English, German and Italian) *[border with Austria region of Italian Alps]	FNS WTC Chontacuros (*Rhynchophorus palmarum L.):* an insect species considered a delicacy amongst indigenous people and settlers of the Ecuadorian Amazon rainforest.	FNS negatively influences WTC. Persuasion strategies positively influence WTC.	[12]
North Italy (Milan) university students, employees and consumers from outside the university (n.341: 223 females and 118 males—18–80 years)	Questionnaire: *willingness to incorporate insects into diets* Subjects divided in 3 groups: “low neophobia” (FNS scores ≤ 23, n=86), “medium neophobia” (FNS scores ≥ 24 and ≤ 41, n=166) “high neophobia” (FNS scores ≥ 42, n=89)	People with *Low level of food neophobia* were significantly more willing to accept insects as feed, as food and served in an ethnic restaurant than people with a *medium level of food neophobia* who, in turn, showed a significantly higher readiness than *neophobic consumers*. Younger people more readily accepted insects. University students and staff (e.g., High level of education) more readily accepted insects. Environmental and nutritional benefits marginally affected the acceptability of insect-based foods.	[73]
North Italy (Milan) and South Italy (Bari) university students (n.35561, 69% female, 18–29 years	Online questionnaires concerning the WTT different food containing insect or earthworm ingredients	38% more likely to consider insect- food part of Italian diet, 32% rejected. Insects/earthworms more accepted in salty snacks. Gender influenced WTT.	[74]
North Italy (Padua) university students with part-time occupation (72%) plus employees in several jobs (n.32—20–35 years)	WTE Insect-based food: cheddar cheese larvets, lollipops, chocolate covered scorpions, salt infused with chili and agave worms, dried crickets, baked grasshoppers, toasted scorpions	WTE is dependent on the form in which the products are presented. Crustaceans were frequently mentioned as a comparison in terms of distaste.	[75]
North Italy (Parma) (n.46 individuals recruited at a “bug banquet”)	The nutritional and the environmental benefits of entomophagy were explained Tasting insects:-House cricket (*Acheta domesticus*) -Wax moth larvae (*Galleria mellonella*) -Grasshoppers (*Calliptamus italicus*)	WTT is determined by curiosity and disgust. 63% of the sample who tasted the insects preferred wax moth larvae, followed by locusts (19%) and crickets (12%); 6% indicated that none of the three species above were preferred.	[76]
North Italy (Parma) university students (n.109, 53% females, 18–25 years)	WTT “Bug tasting session”: Cookie product made by replacing 10% of the traditional flour with “cricket flour” (*Acheta domesticus*).	47% believed that entomophagy might become a culinary trend in Italy, whilst the other half states that it would not be “successful”, “appropriate” or “exciting. 67.5% indicated they would taste edible insects if they had the opportunity. “Bug-tasting session”: 94% of the students agreed to eat the insect-based food.	[77]
North Italy (Parma) university students (n.231, 62% female, mean age 23.6 ± 3.8 years.	ITE Tasting: chocolate chip cookie containing 10% of cricket flour (*Acheta domesticus*)	Weak ITE products containing insect flour. Only 110 individuals ITE and only 53 (22.9% of the total) students tasted the novel food.	[78,79]
North Italy (Parma): university students and staff (n.88, 45 females,18–40 years from: 20% North East, 36% North West, 14% Central and 30% Southern Italy).	FNS, WTT. Tasting two sweet jellies: one with a visible cricket (unprocessed) and one with a processed cricket.	WTT is affected by the FNS. Males were more WTT new foods. WTT-unprocessed < WTT-processed insect-based product. 75% tasted both products 19% tasted only the insect-based jelly 6% did not try either product	[11,80,81]
Central Italy (Pisa) university students (n.165)	Informative seminar entitled “Insects as Food and Feed: Future Prospects” n. 66 [40%] took part of a tasting session: two bread samples identical, except one was claimed to be supplemented with insect powder, e.g., “insect-labelled” bread, although it did not contain any insect ingredients.	No gender impact. WTT is positively affected by behavioural intention. The belief of positive effects on health has a stronger influence on behavioural intentions when compared to beliefs about environmental protection and familiar taste. After the seminar, disgust factor and the fear of negative texture properties was reduced.	[82]
Central Italy (Viterbo) (n.201, female: 55%, mean age 43 years) Education: 19%lower, 49%secondary, 32%university	Insect pictures were showed: Insect-based preparation comparable to sushi Street food stand with fried insects Skewers with pupae Plate with larvae and pupae with some vegetables Meat burger with some larvae on the top	31% WTT insects as food 5% had already tried insects The fear of insects and the idea that the taste might be disgusting were the main barriers to the WTT Familiarity with foreign food, higher education and gender (male) positively influenced consumer attitude to entomophagy.	[83]
South Italy (Naples) university students (n.118: 58 females and 60 males)	Computer questionnaire “Insects vs. flowers” FNS Disgust sensitivity scale	FNS significantly correlates with intention but not with disgust. Intention correlated significantly with disgust.	[84]
South Italy (Naples) (n.200 university students, 40% female, 18–20 years)	General information on benefits on health and environment were given. WTP of 3 categories of foods (pasta, cookies and chocolate bars) with insects (and their conventional counterparts were evaluated).	Without information: - insect-based products lower WTP in the case of cookies and chocolate - same WTP for the two versions of pasta With information on benefits: consumers’ WTP increase for all the products. Food Neophobia negatively affected the WTP for insect-based products	[85]
South Italy (Naples) students or just graduates (n.45)	Drivers of acceptance to insects.	Curiosity drives acceptance. Disgust and food neophobia were related to low acceptance.	[86]
Italians (n.135 individuals, 46% female, 18–35 years)	Insect pictures were showed: Flour/whole insects Opaque/transparent packaging Cacao flavor High protein content (30% of protein content) Environmental certification	Flour-based products WTB > whole insect products. No different WTB: - Transparent vs. opaque packaging - cacao flavor (Food Neophobia increased WTB for cacao flavor presence) - high protein claim - environmental certification	[87]
Italians (n.128) Danish (n.136)	Communication of societal benefits and individual benefits ITE	Communication increased ITE. Danish participants had higher ITE than Italians.	[60]
Italian (n.543) Danish (n.975)	EAQ (EAQ-I; EAQ-D; EAQ-F) Disgust Scale FNS WTE	Negative relation EAQ-F vs. WTE, found only in the Danish population. Danish: EAQ-I main predictor. Regression coefficient EAQ-D vs. WTE is much smaller than that of EAQ-I. Italians: no such great difference between predictive power of EAQ-D and EAQ-I on WTE.	[88]

EAQ=entomophagy attitude questionnaire; EAQ-I=interest subscale of EAQ; EAQ-D=disgust subscale of EAQ; EAQ-F=feeding animals subscale of EAQ; FNS=food neophobia scale; ITE: intention to eat; WTE: willingness to eat; WTC: willingness to consume; WTT=willingness to try; WTP=willingness to pay; WTB=willingness to buy.
